# Clinicopathologic Characteristics of Breast Cancer According to the Infiltrating Immune Cell Subtypes

**DOI:** 10.3390/ijms21124438

**Published:** 2020-06-22

**Authors:** Hye Min Kim, Ja Seung Koo

**Affiliations:** Department of Pathology, Yonsei University College of Medicine, Seoul 03722, Korea; pinkmin15@yuhs.ac

**Keywords:** breast cancer, tumor microenvironment, infiltrating immune CD8 cells, prognosis

## Abstract

The clinical significance of immune cell subtypes in breast cancer remains poorly understood. To identify tumor-infiltrating immune cell subtypes in breast cancer and investigate their implications, tissue microarrays were constructed using 334 cases of invasive ductal carcinoma (luminal A type: 162 (48.5%), luminal B type: 96 (28.7%), HER-2 type: 21 (6.3%), and triple negative breast cancer: 55 (16.5%)). Hormone receptors (ER, PR, and HER-2), Ki-67, and immune cell subtype-related proteins (STAT4, STAT6, FOXP3, CD8, CD68, and CD163) were assessed immunohistochemically. The proportion of highly expressed STAT6, FOXP3, CD8, CD68, and CD163 proteins was found to be lowest in luminal A type but highest in the HER-2 type. Additionally, high-level STAT6, FOXP3, CD68, and CD163 protein expression was associated with higher histologic grade. ER negativity was associated with high STAT6, FOXP3, and CD163 expression levels, whereas PR negativity and high Ki-67 labeling index were associated with high CD163 expression. Univariate (*p* = 0.003) and multivariate Cox (hazard ratio: 2.435, 95% CI: 1.110-5.344, *p* = 0.049) analyses showed that high CD8 expression is an independent factor associated with shorter disease-free survival. Immune cell subtype-related protein expression is dependent on breast cancer molecular subtypes, and CD8 expression is associated with patient prognosis.

## 1. Introduction

Stromal cells, comprising the tumor microenvironment (TME), can be divided into the following types: cancer-associated fibroblast, angiogenic vascular cells, and infiltrating immune cells [1]. These components influence tumor growth and progression through various cross-talks with cancer cells. The infiltrating immune cells are comprised of dendritic cells, T-cells, macrophages, myeloid derived suppressor cells, and natural killer cells [2]. Accumulating evidence emphasizes that tumor infiltrating lymphocytes (TILs), including naïve T cells, effector T cells, and memory T cell subtypes plays a crucial role in the TME by exerting anti-tumor activity, whereas tumor-associated macrophages are responsible in promoting cancer progression [3,4,5]. Even though various markers have been proposed to assess T cells and macrophages, it has been traditionally regarded that STAT4, STAT6, FOXP3, and CD8 represents T helper (Th) 1, Th 2, regulatory T cell, cytotoxic T cell related marker, while CD68 and CD163 are suggested to be a marker of M1 and M2 macrophages [6,7,8,9,10].

Breast cancer is a histologically heterogenous tumor that typically presents with immune cell deposition in the TME. It is noteworthy that the WHO (World Health Organization) classification of tumors classifies individual histological subtypes with high immune cell infiltration as medullary carcinoma, atypical medullary carcinoma, or invasive carcinoma with medullary features [11]. Specifically, the molecular subtypes of breast cancer that are reported to be associated with abundant immune cell infiltration are HER-2 rich type and basal-like type/triple negative breast cancer [12]. In a previous study, TIL was shown to be associated with complete chemotherapy responses in HER-2-positive and triple negative breast cancer [13]. Moreover, TIL was found to be related to longer survival after adjuvant chemotherapy in TNBC [14]. Additionally, a study by Liu et al. demonstrated that the infiltration of CD8^+^ lymphocytes is an independent prognostic factor in basal-like breast cancer [15]. Therefore, although TILs are thought to play an important role in breast cancer, previous studies on TIL have only focused on evaluating phenotypes using CD markers of CD4 and CD8 in invasive ductal carcinoma. Moreover, even though there are studies that investigated markers of FOXP3, CD163, and CD68, investigations have been only performed in pathologic subtypes of ductal carcinoma in situ, and the clinical relevance of immune cell subtypes has been poorly studied [15,16,17,18,19,20]. Therefore, the purpose of this study was to identify subtypes of tumor-infiltrating immune cells in breast cancer and to investigate their implications.

## 2. Results

### 2.1. Basal Characteristics of Breast Cancer Patients

Of the 334 cases studied, 162 (48.5%), 96 (28.7%), 21 (6.3%), and 55 (16.5%) cases were luminal A, luminal B, HER-2 type, and triple negative breast cancer, respectively. The clinicopathologic features based on the breast cancer molecular subtypes are shown in Supplementary Table S1. The histologic grade was significantly different based on the molecular subtype (*p* < 0.001); however, there was no difference based on age, tumor stage, and nodal stage. The ratio of lymphocyte predominant breast cancer (LPBC) was significantly lower in luminal A type compared to those in other subtypes (*p* < 0.001).

### 2.2. Expression of Immune Cell Subtype-Related Proteins According to the Breast Cancer Molecular Subtype

**Figure 1:**
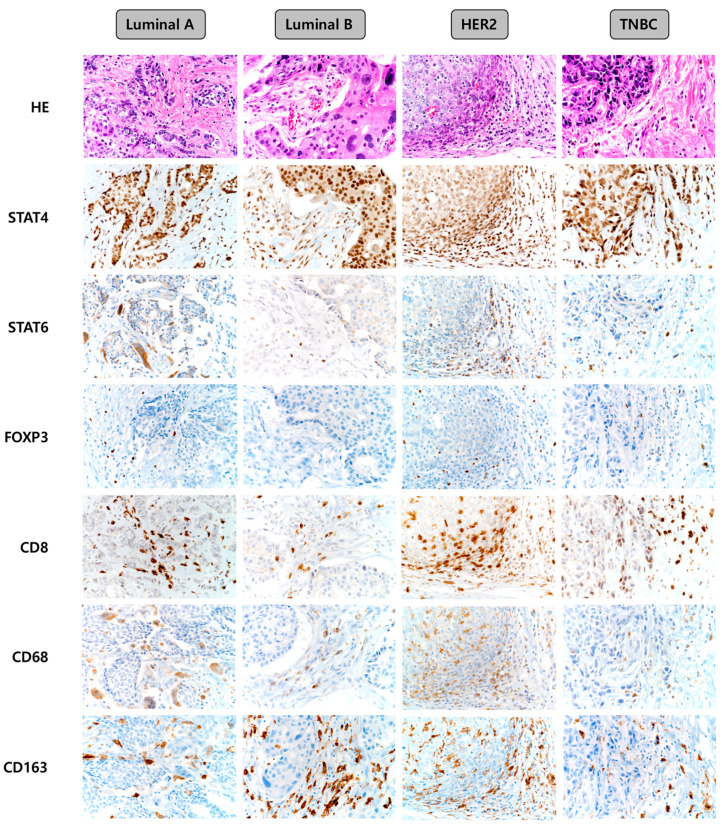
Representative images of immune cell subtype-related protein expression according to the breast cancer molecular subtypes. TNBC, triple-negative breast cancer.

Representative images of the expression of immune cell subtype-related proteins according to the breast cancer molecular subtype are shown in Figure 1. The median values of the expression levels of immune cell subtype-related proteins were as follows: STAT4 h-score: 90, STAT6 h-score: 0, FOXP3: 0, CD8: 5, CD68: 0, and CD163: 10. Next, we classified the values below and above the median level as low and high expression, respectively, and compared the expression of each molecule among the breast cancer molecular subtypes. Consequently, the frequencies of high expression of STAT6 (*p* = 0.047), FOXP3 (*p* = 0.006), CD8 (*p* < 0.001), CD68 (*p* = 0.026), and CD163 (*p* < 0.001) proteins were found to be lowest in the luminal A type, while high expression of these proteins was more frequent in the HER-2 type (Table 1). On comparing the number of immune cells expressing immune cell subtype-related proteins, we found that the expression of STAT6 (*p* = 0.005), FOXP3 (*p* = 0.001), CD8 (*p* = 0.012), CD68 (*p* = 0.011), and CD163 (*p* < 0.001) differed significantly according to the breast cancer molecular subtypes. The proportions of immune cells expressing STAT6, FOXP3, CD8, CD68, and CD163 were the lowest in luminal A and highest in HER-2 type (Table 2).

Among the 334 cases, the proportion of highly expressed immune cell subtype-related proteins was significantly higher in LPBC cases (*p* < 0.05). In an LPBC subgroup analysis, and according to the molecular subtype, we observed a significantly higher proportion of highly expressed CD8 (*p* < 0.001), CD68 (*p* = 0.003), and CD163 (*p* < 0.001) in luminal A type; STAT6 (*p* = 0.016), CD8 (*p* < 0.001), CD68 (*p* = 0.002), and CD163 (*p* = 0.001) in luminal B type; STAT4 (*p* = 0.041) and CD8 (*p* = 0.002) in HER-2 type; and STAT4 (*p* = 0.021), CD8 (*p* = 0.005), CD68 (*p* = 0.023), and CD163 (*p* = 0.023) in TNBC (Table 3).

### 2.3. Correlations among the Expression Statuses of Immune Cell Subtype-Related Proteins

Significant positive correlations in expression statuses were observed among the immune cell subtype-related proteins (Supplementary Table S2) as follows: STAT4–STAT6 (*r* = 0.189, *p* = 0.001), STAT4–FOXP3 (*r* = 0.229, *p* < 0.001), STAT4–CD8 (*r* = 0.293, *p* < 0.001), STAT4–CD68 (*r* = 0.140, *p* = 0.010), STAT4–CD163 (*r* = 0.200, *p* < 0.001), STAT6–FOXP3 (*r* = 0.303, *p* < 0.001), STAT6–CD8 (*r* = 0.268, *p* < 0.001), STAT6–CD68 (*r* = 0.276, *p* < 0.010), STAT6–CD163 (*r* = 0.355, *p* < 0.001), FOXP3–CD8 (*r* = 0.176, *p* = 0.001), FOXP3–CD68 (*r* = 0.295, *p* < 0.001), FOXP3–CD163 (*r* = 0.321, *p* < 0.001), CD8–CD68 (*r* = 0.447, *p* < 0.001), CD8–CD163 (*r* = 0.463, *p* < 0.001), and CD68–CD163 (*r* = 0.485, *p* < 0.001).

### 2.4. Correlation between Clinicopathologic Parameters and the Expression Statuses of Immune Cell Subtype-Related Proteins

The correlations between the proportions (%) of ER and PR expression and the expression levels of immune cell subtype-related proteins (H-score or %) were investigated. ER and PR expression, respectively, showed significant negative correlation with the expression of FOXP3 (*r* = −0.197, *p* < 0.001 and *r* = −0.156, *p* = 0.004), CD68 (*r* = −0.131, *p* = 0.017 and *r* = −0.149, *p* = 0.006), and CD163 (*r* = −0.298, *p* < 0.001 and *r* = −0.204, *p* < 0.001). Stromal TIL (%) and the expression levels of immune cell subtype-related proteins (H-score or %) were significantly positively correlated for all immune cell subtype-related proteins (Table 4).

**Figure 2:**
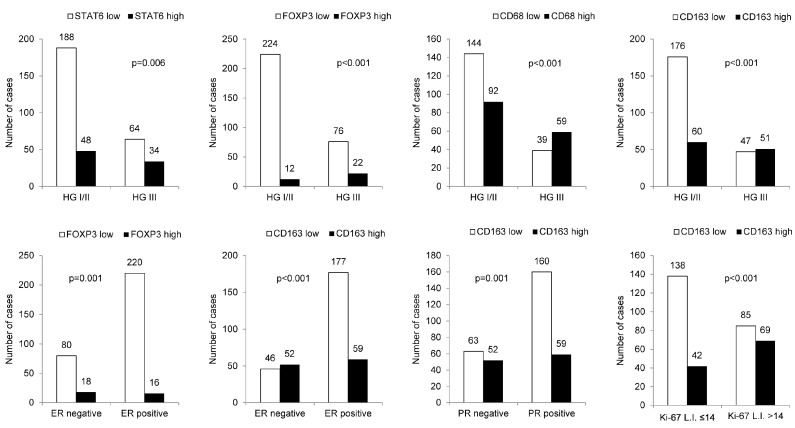
The relationship between clinicopathologic parameters and immune cell subtype-related protein expression. A higher histologic grade was associated with high expression levels of STAT6, FOXP3, CD68, and CD163; ER negativity was associated with high STAT6, FOXP3, and CD163 expression levels, whereas PR negativity and high Ki-67 L.I. were associated with high CD163 expression. HG: histologic grade; L.I.: labeling index.

When the relationships between clinicopathologic parameters with high- and low-expression of immune cell subtype-related proteins were assessed, higher histologic grade was associated with high STAT6, FOXP3, CD68, and CD163 expression levels. Furthermore, ER negativity was associated with high STAT6, FOXP3, and CD163 expression levels, whereas PR negativity and high Ki-67 labeling index (L.I.) were associated with high CD163 expression (Figure 2).

### 2.5. Impact of Immune Cell Subtype-Related Protein Expression on Patient Prognosis

**Figure 3:**
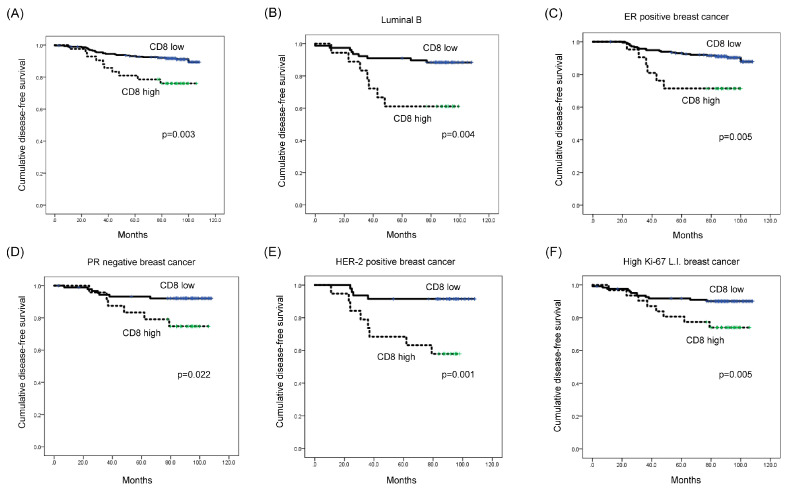
Impact of immune cell subtype-related protein expression on patient prognosis (disease-free survival, DFS). (**A**) High-level CD8 expression was associated with shorter DFS (*p* = 0.003). (**B**–**F**) High-level CD8 expression was associated with shorter DFS in luminal B type (*p* = 0.004), ER-positive (*p* = 0.005), PR-negative (*p* = 0.022), HER-2-positive (*p* = 0.001), and high Ki-67 L.I. (Ki-67 L.I. > 14; *p* = 0.005) breast cancer. L.I.: labeling index.

Univariate Cox-proportional hazard analysis showed that a high CD8 expression level was associated with shorter disease-free survival (DFS) (*p* = 0.003; Table 5 and Figure 3A). Additionally, multivariate analysis revealed that a high CD8 expression level was an independent factor associated with shorter DFS (hazard ratio: 2.435, 95% CI: 1.110-5.344, *p* = 0.049; Table 6).

**Figure 4:**
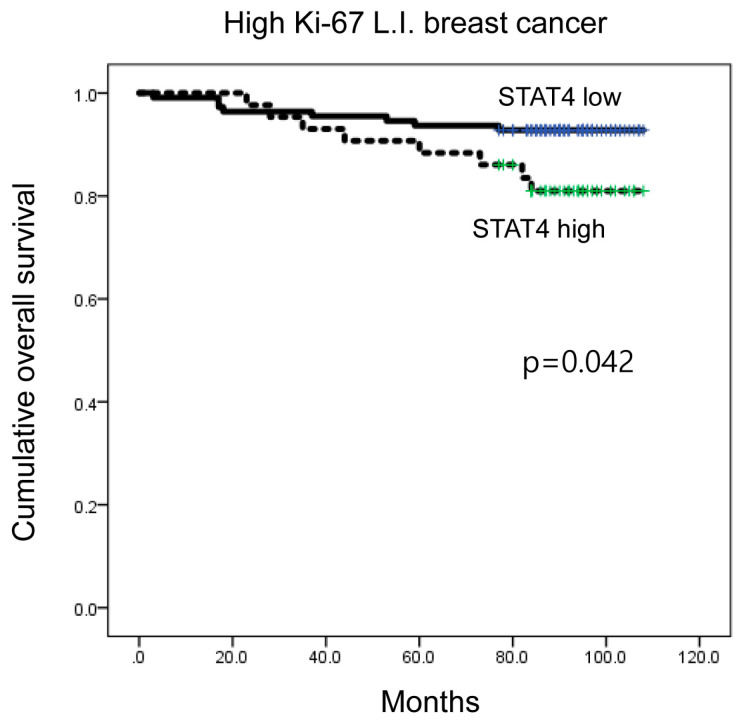
Impact of immune cell subtype-related protein expression on patient prognosis (overall survival, OS). In high Ki-67 L.I. breast cancer, high STAT4 expression was associated with shorter OS (*p* = 0.042). L.I.: labeling index.

When a subgroup analysis was performed, high CD8 expression was associated with shorter DFS in luminal B type (*p* = 0.004; Figure 3B), as well as in ER-positive breast cancer (*p* = 0.005) (Figure 3C). Moreover, high CD8 expression was associated with shorter DFS in PR-negative, HER-2-positive, and high Ki-67 L.I. (Ki-67 L.I. > 14) breast cancers (*p* = 0.022, *p* = 0.001, and *p* = 0.005, respectively; Figure 3D–F). In high Ki-67 L.I. breast cancer, high-level STAT4 expression was associated with shorter overall survival (OS) (*p* = 0.042; Figure 4).

## 3. Discussion

In this study, we aimed to identify subtypes of tumor-infiltrating immune cells in breast cancer and to investigate their implications. We found that the proportion of highly expressed STAT6, FOXP3, CD8, CD68, and CD163 proteins was the lowest in the luminal A type, but more frequent in the HER-2 type. Previous studies have reported that stromal TILs are less frequently found in the luminal type than in the HER-2 type and TNBC [21,22,23,24]. Additionally, the proportion of cases with LPBC, which is defined as cancer with >50% stromal TILs, was also reported to be lower among the luminal type and higher among the HER-2 type and TNBC [22,23,25,26,27], which was identically observed in this study. Therefore, it can be said that in the luminal type, which has a small number of absolute immune cells, the number of immune cells that are positive for immune cell subtype-related proteins is low. Additionally, an analysis based on the molecular subtypes showed that the number of immune cells expressing CD8, CD68, and CD163 was higher in LPBC of luminal A type, indicating that the total number of stromal immune cell is an important determinant of immune cell subtype-related protein expression. However, among HER-2 type and TNBC, which generally have higher stromal TIL % and LPBC, the proportion of highly expressed STAT6, FOXP3, CD8, CD68, and CD163 in HER-2 was observed to be high, and was found to be statistically significant for CD8 (*p* = 0.008). Based on these results, it can be suggested that HER-2 type and TNBC have different immune cell subtypes, even though they both present with increased stromal immune cell infiltration.

In a systematic review that analyzed infiltrating immune cell subtypes in breast cancer, CD8 was reported to exist similarly in TNBC and HER-2-positive breast cancer [27]. However, given that CD8 was included as a dichotomous variable (in absence/presence) in a study by Stanton et al. and that hormone receptor positive HER-2-positive breast cancer was included within HER-2-positive breast cancer, it seemed difficult to compare the results directly. Yet, in the case of FOXP3, when the results were analyzed by classification into low and high groups, high FOXP3 expression was reported in 70% of TNBC and 67% of HER-2-positive breast cancer, which is similar to our study [27]. Nevertheless, as this study also included hormone receptor positive HER-2-positive breast cancer, the difference in the study groups should also be taken into consideration. Likewise, another study demonstrated that the number of FOXP3 and CD8 positive stromal TILs was higher in HER-2-positive breast cancer than TNBC in the invasive margin, showing similar results with this study [28]. However, Papaioannou et al. reported a contradictory result, indicating no difference in the T-cell subtype of TILs according to the molecular subtype, an observation that should be evaluated in further studies [29].

Considering the higher expression of the cytotoxic T-cell marker CD8 in HER-2-positive breast cancer compared to that in TNBC, a possible relationship between HER-2 amplification and T cell immunity could exist. As the *HER-2* oncogene contains several major histocompatibility complex class I-restricted epitopes, it can be recognized by cytotoxic T lymphocytes (CTLs), such as CD8. Therefore, in HER-2 amplified cancer, many immune reactions could be present due to CTL infiltration of affected tissues [30,31].

Interestingly, we found that the expression of the tumor-associated macrophage (TAM) markers CD68 and CD163 was higher in HER-2-positive breast cancer. Although this association is not well studied [32,33,34,35,36], in an experiment that was performed using an animal model of HER-2-positive breast cancer, CCL2, which is produced by cancer and myeloid cells, attracted macrophages to upregulate Wnt-1 and downregulate E-cadherin, resulting in cancer dissemination. Additionally, HER-2 or another oncogenic signal could be responsible for the activation of mammary epithelial cells and macrophages [37,38,39,40], which seems to be relevant to the higher expression of TAM markers in HER-2-positive breast cancer.

Regarding patient prognosis, a high CD8 expression level was found to be an independent poor prognostic factor in all breast cancers and was associated with shorter DFS in luminal B, ER-positive, PR-negative, HER-2-positive, and high Ki-67 L.I. breast cancer. Although a previous study reported a contradictory result that increased CD8^+^ cells in TNBC were associated with better prognosis [15], the discordant result could be related to the fact that we investigated stromal CD8^+^ immune cells, while the previous study investigated intratumoral CD8^+^ immune cells. Moreover, whether intratumoral CD8^+^ immune cells are a positive or negative prognostic factor remains unclear, as a study by Catacchio et al. has shown that intratumoral CD8^+^ immune cells are a negative prognostic factor in all breast cancers [41]. The association between CD8^+^ immune cells and breast cancer prognosis can be influenced by multiple factors, such as density, localization, and spatial distribution in TME [42]. Since we used tissue microarray to analyze tumor-infiltrating immune cells in this study, rather than whole sections for histological examination, samples may have been influenced by extraction bias during tissue microarray construction and may not be sufficient in assessing tumor heterogeneity. In addition, as the number of cases included in this study was relatively small, this could have also affected in the interpretation of study results. Thus, the additional studies are necessary for comprehensive evaluation and verification.

The clinical significance of this study is that TIL assessment could have an important implication in breast cancer treatment. For instance, it has been reported that TIL predicts responsiveness to specific therapeutic agents in breast cancer and predicts targeted therapy efficacy in specific molecular subtypes of breast cancer [43]. Therefore, there is a possibility that TIL could be utilized as a candidate biomarker for immune therapy, which is currently emphasized in breast cancer treatment. Recently, the expression of PD-L1, an important immune therapy biomarker, has been associated with TIL in breast cancer [44,45]. Moreover, PD-L1 infiltration was reported to be correlated with CD8^+^ immune cell infiltration in TNBC [46]. Importantly, when CD8^+^ immune cells are present in TIL and PD-L1^+^ immune cells are present in breast cancer, immune checkpoint blockade has shown therapeutic benefit [47,48].

Taken together, our results successfully demonstrated that the expression of the immune cell subtype-related proteins STAT6, FOXP3, CD8, CD68, and CD163 was different according to the molecular subtype of breast cancer. In particular, CD8 expression was found to be a prognostic factor. Our results strongly suggest that investigating TIL subgroups could provide useful prognostic and therapeutic information in breast cancer. However, future investigations are necessary for evaluating a possible difference in clinical responses to immune therapy depending on immune cell marker expression.

## 4. Materials and Methods

### 4.1. Patient Selection and Histologic Evaluation

This study was performed using specimens from patients diagnosed with invasive ductal carcinoma after surgical removal of breast tissue at Yonsei University Severance Hospital, Seoul, South Korea from January 2000 to December 2006. Patients who received chemotherapy or hormone therapy prior to the surgery were excluded. This study was approved by the Institutional Review Board of Yonsei University Severance Hospital (14 November 2016; 4-2016-0832), which exempted informed consent from patients. All cases were reviewed by an experienced breast pathologist (Koo JS) using Hematoxylin & Eosin (H & E)-stained slides. Histological grading was assessed using the Nottingham grading system [49]. LPBC was defined as more than 50% of stromal TIL, as previously described [50]. Clinicopathologic parameters evaluated included patient age at initial diagnosis, lymph node metastasis, tumor recurrence, distant metastasis, and patient survival.

### 4.2. Tissue Microarray

Tissue microarrays were constructed using formalin-fixed and paraffin-embedded (FFPE) tissues from surgically resected breast cancer. Representative areas from the donor block were selected, extracted using a 3-mm-core punch machine, and placed in the donor block; cores with >30% of the tumor cells were considered valid cores.

### 4.3. Immunohistochemistry

Immunohistochemistry (IHC) was performed using a BenchMark automated staining instrument (Ventana Medical System, Tucson, AZ, USA). Primary antibodies used for IHC staining are listed in Supplementary Table S3. Tissue samples were sectioned to 4 µm thickness, deparaffinized in xylene, rehydrated in three graded alcohol chambers, and treated with 3% hydrogen peroxide in methanol. For stain visualization, a 3,3′-Diaminobenzidine (DAB) detection kit (Ventana Medical System, Tucson, AZ, USA) was used.

### 4.4. Immunohistochemistry Interpretation

Immunohistochemical staining assessment via tissue microarrays was performed independently by two pathologists (Koo JS and Kim HM), as previously reported [51,52]. TILs that were positive for each immunohistochemical marker were scored for the stromal compartment. The percentage of stromal TILs was determined by dividing the area occupied by stromal TILs by the entire stromal tissue area (i.e., area possessed by mononuclear inflammatory cells divided by total intratumoral stromal area). TILs were evaluated within the borders of the invasive tumor and all mononuclear cells (including lymphocytes and plasma cells) were scored.

### 4.5. Breast Cancer Molecular Classification

We classified breast cancer phenotypes based on the IHC results for the hormone receptors (ER, PR, and HER-2) and Ki-67, and FISH results for HER-2 as follows [53]: *luminal A type*, ER- or/and PR-positive, HER-2-negative, and Ki-67 L.I. < 14%; *Luminal B type*, (HER-2-negative) ER- or/and PR-positive, HER-2-negative, and Ki-67 L.I. ≥ 14%; (HER-2-positive) ER- or/and PR-positive and HER-2 overexpressed or/and amplified; *HER-2 type*, ER- and PR-negative and HER-2 overexpressed or/and amplified; and *TNBC type*: ER-, PR-, and HER-2-negative.

### 4.6. Statistical Analysis

Data were analyzed using IBM SPSS Statistics for Windows, Version 21.0 (IBM Corp. Released 2012. Armonk, NY, USA). The χ^2^ or Fisher’s exact test was used to compare the expression of tumor-infiltrating immune cells between breast cancer subgroups, as appropriate. A corrected *p*-value, determined using the Bonferroni multiple comparison procedure, was used for multiple comparisons. Statistical significance was set to *p* < 0.05. Kaplan-Meier survival curves and log-rank test were employed to compare DFS and OS rates. Univariate and multivariate regression analyses were performed using the Cox proportional hazards model.

## Figures and Tables

**Table 1:** Expression of immune cell subtype-related proteins according to the breast cancer molecular subtype (Low versus High).

Parameter	Total (*n* = 334) (%)	Luminal A (*n* = 162) (%)	Luminal B (*n* = 96) (%)	HER-2 (*n* = 21) (%)	TNBC (*n* = 55) (%)	*p*-Value
STAT4						0.809
Low	237 (71.0)	115 (71.0)	71 (74.0)	14 (66.7)	37 (67.3)	
High	97 (29.0)	47 (29.0)	25 (26.0)	7 (33.3)	18 (32.7)	
STAT6						0.047
Low	252 (75.4)	131 (80.9)	65 (67.7)	13 (61.9)	43 (78.2)	
High	82 (24.6)	31 (19.1)	31 (32.3)	8 (38.1)	12 (21.8)	
FOXP3						0.006
Low	300 (89.8)	151 (93.2)	88 (91.7)	15 (71.4)	46 (83.6)	
High	34 (10.2)	11 (6.8)	8 (8.3)	6 (28.6)	9 (16.4)	
CD8						<0.001
Low	292 (87.4)	155 (95.7)	78 (81.3)	12 (57.1)	47 (85.5)	
High	42 (12.6)	7 (4.3)	18 (18.8)	9 (42.9)	8 (14.5)	
CD68						0.026
Low	183 (54.8)	101 (62.3)	48 (50.0)	7 (33.3)	27 (49.1)	
High	151 (45.2)	61 (37.7)	48 (50.0)	14 (66.7)	28 (50.9)	
CD163						<0.001
Low	223 (66.8)	131 (80.9)	57 (59.4)	8 (38.1)	27 (49.1)	
High	111 (33.2)	31 (19.1)	39 (40.6)	13 (61.9)	28 (50.9)	

TNBC: triple-negative breast cancer.

**Table 2:** Expression of immune cell subtype-related proteins according to the breast cancer molecular subtype (H-score or %).

Parameter	Total (*n* = 334) (Mean ± SD)	Luminal A (*n* = 162) (Mean ± SD)	Luminal B (*n* = 96) (Mean ± SD)	HER-2 (*n* = 21) (Mean ± SD)	TNBC (*n* = 55) (Mean ± SD)	*p*-Value
STAT4 H-score	87.7 ± 56.6	87.8 ± 53.6	81.8 ± 55.3	102.8 ± 65.0	92.0 ± 64.0	0.417
STAT6 H-score	2.6 ± 7.3	1.5 ± 3.9	3.5 ± 8.1	7.1 ± 16.3	2.7 ± 7.9	0.005
FOXP3 (%)	0.5 ± 1.5	0.3 ± 1.2	0.4 ± 1.3	1.6 ± 2.8	0.8 ± 1.8	0.001
CD8 (%)	6.3 ± 7.9	5.2 ± 5.3	7.6 ± 9.6	10.2 ± 8.1	6.1 ± 10.1	0.012
CD68 (%)	1.0 ± 1.9	0.7 ± 1.6	1.0 ± 1.7	1.4 ± 2.4	1.7 ± 2.7	0.011
CD163 (%)	14.6 ± 15.6	9.7 ± 11.7	17.4 ± 17.1	22.1 ± 17.4	21.4 ± 17.9	<0.001

TNBC: triple-negative breast cancer; SD: standard deviation.

**Table 3:** Expression of immune cell subtype-related proteins in molecular subtypes of breast cancer based on LPBC status.

Parameter	Total breast cancer			Luminal A			Luminal B			HER-2			TNBC		
	No LPBC *n* = 292 (%)	LPBC *n* = 42 (%)	*p*-Value	No LPBC *n* = 154 (%)	LPBC *n* = 8 (%)	*p*-Value	No LPBC *n* = 81 (%)	LPBC *n* = 15 (%)	*p*-Value	No LPBC *n* = 13 (%)	LPBC *n* = 8 (%)	*p*-Value	No LPBC *n* = 44 (%)	LPBC *n* = 11 (%)	*p*-Value
STAT4			<0.001			0.171			0.060			0.041			0.021
Low	218 (74.7)	19 (45.2)		111 (72.1)	4 (50.0)		63 (77.8)	8 (53.3)		11 (84.6)	3 (37.5)		33 (75.0)	4 (36.4)	
High	74 (25.3)	23 (54.8)		43 (27.9)	4 (50.0)		18 (22.2)	7 (46.7)		2 (15.4)	5 (62.5)		11 (25.0)	7 (63.6)	
STAT6			<0.001			0.180			0.016			0.310			0.182
Low	228 (78.1)	24 (57.1)		126 (81.8)	5 (62.5)		59 (72.8)	6 (40.0)		7 (53.8)	6 (75.0)		36 (81.8)	7 (63.6)	
High	64 (21.9)	18 (42.9)		28 (18.2)	3 (37.5)		22 (27.2)	9 (60.0)		6 (46.2)	2 (25.0)		8 (18.2)	4 (36.4)	
FOXP3			0.010			0.510			0.107			0.410			0.582
Low	267 (91.4)	33 (78.6)		144 (93.5)	7 (87.5)		76 (93.8)	12 (80.0)		10 (76.9)	5 (62.5)		37 (84.1)	9 (81.8)	
High	25 (8.6)	9 (21.4)		10 (6.5)	1 (12.5)		5 (6.2)	3 (20.0)		3 (23.1)	3 (37.5)		7 (15.9)	2 (18.2)	
CD8			<0.001			<0.001			<0.001			0.002			0.005
Low	279 (95.5)	13 (31.0)		152 (98.7)	3 (37.5)		75 (92.6)	3 (20.0)		11 (84.6)	1 (12.5)		41 (93.2)	6 (54.5)	
High	13 (4.5)	29 (69.0)		2 (1.3)	5 (62.5)		6 (7.4)	12 (80.0)		2 (15.4)	7 (87.5)		3 (6.8)	5 (45.5)	
CD68			<0.001			0.003			0.002			0.112			0.023
Low	177 (60.6)	6 (14.3)		100 (64.9)	1 (12.5)		46 (56.8)	2 (13.3)		6 (46.2)	1 (12.5)		25 (56.8)	2 (18.2)	
High	115 (39.4)	36 (85.7)		54 (35.1)	7 (87.5)		35 (43.2)	13 (86.7)		7 (53.8)	7 (87.5)		19 (43.2)	9 (81.8)	
CD163			<0.001			<0.001			0.001			0.310			0.023
Low	215 (73.6)	8 (19.0)		130 (84.4)	1 (12.5)		54 (66.7)	3 (20.0)		6 (46.2)	2 (25.0)		25 (56.8)	2 (18.2)	
High	77 (26.4)	34 (81.0)		24 (15.6)	7 (87.5)		27 (33.3)	12 (80.0)		7 (53.8)	6 (75.0)		19 (43.2)	9 (81.8)	

TNBC: triple-negative breast cancer; LPBC: lymphocyte predominant breast cancer.

**Table 4:** Correlations between the proportions (%) of ER, PR, and stromal TIL expression levels and immune cell subtype-related protein expression levels.

Parameters	STAT4 H-Score	STAT6 H-Score	FOXP3	CD8	CD68	CD163
ER						
*r*-coefficient	−0.086	−0.148	−0.197	−0.046	−0.131	−0.298
*p*-value	0.117	0.007	<0.001	0.399	0.017	<0.001
PR						
*r*-coefficient	−0.051	−0.083	−0.156	−0.105	−0.149	−0.204
*p*-value	0.357	0.131	0.004	0.055	0.006	<0.001
Stromal TIL						
*r*-coefficient	0.313	0.316	0.175	0.665	0.465	0.525
*p*-value	<0.001	<0.001	0.001	<0.001	<0.001	<0.001

TIL, tumor infiltrating lymphocyte.

**Table 5:** Univariate analysis of the impact of infiltrating immune cell subtype-related protein expression in breast cancers on disease-free survival and overall survival using the log-rank test.

Parameter	Number of Patients /Recurrence/Death	Disease-Free Survival		Overall Survival	
		Mean Survival (95% CI) Months	*p*-Value	Mean Survival (95% CI) Months	*p*-Value
STAT4			0.346		0.074
Low	237/23/17	101 (98–104)		103 (101–105)	
High	97/13/13	98 (93–103)		100 (97–104)	
STAT6			0.995		0.054
Low	252/27/27	100 (97–103)		102 (99–104)	
High	82/9/3	100 (95–105)		105 (102–108)	
FOXP3			0.672		0.957
Low	300/33/27	100 (97–102)		102 (100–104)	
High	34/3/3	101 (94–108)		103 (99–108)	
CD8			0.003		0.475
Low	292/26/25	101 (99–104)		103 (101–105)	
High	42/10/5	90 (81–99)		99 (94–105)	
CD68			0.136		0.629
Low	183/15/15	100 (97–103)		101 (99–104)	
High	151/21/15	99 (95–102)		103 (100–105)	
CD163			0.714		0.681
Low	223/23/19	100 (97–103)		103 (101–105)	
High	111/13/11	100 (95–104)		102 (99–105)	

**Table 6:** Multivariate analysis of the impact of prognostic factors of breast cancers on disease-free survival and overall survival.

Parameters	Disease-Free Survival			Overall Survival		
	Hazard Ratio	95% CI	*p*-Value	Hazard Ratio	95% CI	*p*-Value
Age			0.627			0.021
≤50 versus >50	1.185	0.597–2.353		2.418	1.145–5.108	
Histologic grade			0.220			0.201
I/II versus III	1.674	0.735–3.814		1.784	0.734–4.338	
T stage			0.765			0.816
T1 versus T2/3	0.900	0.451–1.797		0.915	0.436–1.923	
Lymph node metastasis			0.022			0.163
No versus Yes	2.247	1.125–4.488		1.708	0.805–3.626	
ER status			0.162			0.453
Negative versus Positive	0.481	0.173–1.342		1.490	0.526–4.218	
PR status			0.893			0.840
Negative versus Positive	0.943	0.404–2.203		1.096	0.452–2.656	
HER-2 status			0.216			0.450
Negative versus Positive	1.628	0.752–3.525		1.400	0.585–3.348	
Ki-67 L.I.			0.539			0.489
≤14 versus >14	1.258	0.542–2.917		0.705	0.262–1.897	
CD8			0.026			0.975
Low versus High	2.435	1.110–5.344		0.984	0.355–2.728	

L.I., labeling index.

## Supplementary Materials

Supplementary materials can be found at https://www.mdpi.com/1422-0067/21/12/4438/s1.
