# Supplementary material for: Clinicopathologic Characteristics of Breast Cancer According to the Infiltrating Immune Cell Subtypes
Source: Int J Mol Sci. 2020 Jun 22;21(12):4438. doi: 10.3390/ijms21124438 (PMC7352832; doi:10.3390/ijms21124438)
Supplement: Supplementary file 1 [file ijms-21-04438-s001.pdf]

Table S1. Clinicopathologic characteristics of patients according to the breast cancer molecular subtype

Parameter	Total (n =334) (%)	Luminal A (n =162) (%)	Luminal B (n =96) (%)	HER-2 (n =21) (%)	TNBC (n =55) (%)	*p*-Value
Age (years)						0.199
≤50	206 (61.7)	108 (66.7)	54 (56.3)	10 (47.6)	34 (61.8)	
>50	128 (38.3)	54 (33.3)	42 (43.8)	11 (52.4)	21 (38.2)	
Histologic grade						<0.001
I/II	236 (70.7)	153 (94.4)	53 (55.2)	9 (42.9)	21 (38.2)	
III	98 (29.3)	9 (5.6)	43 (44.8)	12 (57.1)	34 (61.8)	
Tumor stage						0.908
T1	184 (55.1)	94 (58.0)	51 (53.1)	10 (47.6)	29 (52.7)	
T2	147 (44.0)	67 (41.4)	44 (45.8)	11 (52.4)	25 (45.5)	
T3	3 (0.9)	1 (0.6)	1 (1.0)	0 (0.0)	1 (1.8)	
Nodal stage						0.215
N0	199 (59.6)	84 (51.9)	59 (61.5)	16 (76.2)	40 (72.7)	
N1	103 (30.8)	59 (36.4)	28 (29.2)	4 (19.0)	12 (21.8)	
N2	24 (7.2)	14 (8.6)	7 (7.3)	1 (4.8)	2 (3.6)	
N3	8 (2.4)	5 (3.1)	2 (2.1)	0 (0.0)	1 (1.8)	
LPBC						<0.001
No	292 (87.4)	154 (95.1)	81 (84.4)	13 (61.9)	44 (80.0)	
Yes	42 (12.6)	8 (4.9)	15 (15.6)	8 (38.1)	11 (20.0)	
Stromal TIL (%)						<0.001
Mean±SD	21.9±31.4	8.8±18.3	19.7±28.5	36.5±40.0	21.9±31.4	
Estrogen receptor status						<0.001
Negative	98 (29.3)	4 (2.5)	18 (18.8)	21 (100.0)	55 (100.0)	
Positive	236 (70.7)	158 (97.5)	78 (81.3)	0 (0.0)	0 (0.0)	
Progesterone receptor status						<0.001
Negative	115 (34.4)	20 (12.3)	19 (19.8)	21 (100.0)	55 (100.0)	
Positive	219 (65.6)	142 (87.7)	77 (80.2)	0 (0.0)	0 (0.0)	
HER-2 status						<0.001
Negative	268 (80.2)	162 (100.0)	51 (53.1)	0 (0.0)	55 (100.0)	
Positive	66 (19.8)	0 (0.0)	45 (46.9)	21 (100.0)	0 (0.0)	
Ki-67 L.I. (%)						<0.001
≤14	180 (53.9)	162 (100.0)	11 (11.5)	1 (4.8)	6 (10.9)	
>14	154 (46.1)	0 (0.0)	85 (88.5)	20 (95.2)	49 (89.1)	

TNBC, triple-negative breast cancer; LPBC, lymphocyte predominant breast cancer; TIL, tumor infiltrating lymphocyte; SD, standard deviation; L.I., labeling index.

Table S2. Correlations in expression statuses among immune cell subtype-related proteins

Parameters	STAT6 H-score	FOXP3	CD8	CD68	CD163
**STAT4 H-score**					
r-coefficient	0.189	0.229	0.293	0.140	0.200
p-Value	0.001	<0.001	<0.001	0.010	<0.001
**STAT6 H-score**					
r-coefficient		0.303	0.268	0.276	0.355
p-Value		<0.001	<0.001	<0.001	<0.001
**FOXP3**					

r-coefficient			0.176	0.295	0.321
p-Value			0.001	<0.001	<0.001
**CD8**					
r-coefficient				0.447	0.463
p-Value				<0.001	<0.001
**CD68**					
r-coefficient					0.485
p-Value					<0.001

Table S3. Source, clone, and dilution of antibodies

Antibody	Company	Clone	Dilution
**Infiltrating immune cell-related proteins**			
STAT4 (Th1 cell related)	Abcam, Cambridge, UK	EP1900Y	1:100
STAT6 (Th2 cell related)	Abcam, Cambridge, UK	YE361	1:50
FOXP3 (Treg related)	Abcam, Cambridge, UK	Polyclonal	1:200
CD8 (cytotoxic T cell related)	Abcam, Cambridge, UK	Polyclonal	1:200
CD68 (M1 related)	Abcam, Cambridge, UK	KP1	1:100
CD163 (M2 related)	Abcam, Cambridge, UK	Polyclonal	1:200
**Molecular subtype-related proteins**			
ER	Thermo Scientific, San Diego, CA, USA	SP1	1:100
PR	DAKO, Glostrup, Denmark	PgR	1:50
HER-2	DAKO, Glostrup, Denmark	Polyclonal	1:1,500
Ki-67	Abcam, Cambridge, UK	MIB	1:1,000
